# The association between grandparents as caregivers and overdose mortality in Appalachia and non-Appalachia counties

**DOI:** 10.3389/fpubh.2023.1035564

**Published:** 2023-02-22

**Authors:** Kate E. Beatty, Stephanie M. Mathis, Abby R. McCurry, Margaret M. Francisco, Michael Meit, Amy E. Wahlquist

**Affiliations:** ^1^Center for Rural Health Research, East Tennessee State University, Johnson City, TN, United States; ^2^Department of Health Services Management and Policy, College of Public Health, East Tennessee State University, Johnson City, TN, United States; ^3^Bill Gatton College of Pharmacy, East Tennessee State University, Johnson City, TN, United States; ^4^Department of Community and Behavioral Health, College of Public Health, East Tennessee State University, Johnson City, TN, United States

**Keywords:** substance abuse and addiction, rural health, grandparent-grandchild contact, social determinants of health, Appalachia

## Abstract

**Objective:**

To assess the association of drug overdose mortality with grandparents serving as caregivers of children in Appalachia and non-Appalachia in the U.S.

**Methods:**

This study used a cross-sectional design, with percent of grandparents as caregivers and overdose mortality rates being of primary interest. County-level data were combined, and descriptive, bivariate, and multivariable statistics were applied. Multiple sociodemographic and geographic variables were included: median age of the population, percent of the population that is uninsured, percent of the population that is non-Hispanic white, teen birth rate, percent of high school dropouts, and rurality.

**Results:**

The percent of grandparents as caregivers increased as the overdose mortality rate increased (*p* < 0.01). For every 1% increase in the overdose mortality rate, the percent of grandparents as caregivers increased by 56% in Appalachian counties compared to 24% in non-Appalachian counties. After adjusting for sociodemographic characteristics, the interaction between overdose mortality and Appalachian vs. non-Appalachian counties was no longer significant (*p* = 0.3).

**Conclusions:**

Counties with higher overdose mortality rates had greater rates of grandparents as caregivers, with Appalachian counties experiencing greater rates of grandparents as caregivers than non-Appalachian counties. Sociodemographic characteristics that are often more prevalent in Appalachia may be driving the observed differences.

**Policy implications:**

Policies and programs are needed to support grandparents providing caregiving for children impacted by substance use disorders including reform to federal child welfare financing to support children, parents, and grandparent caregivers such as kinship navigation, substance use treatment and prevention services, mental health services and in-home supports.

## Introduction

According to the Appalachian Regional Commission (ARC), Appalachia is defined as a region of the United States that consists of 423 counties and eight independent cities (in Virginia) across 13 states from New York to Mississippi (1). Appalachia had ~25.7 million residents in 2019 (2). While the U.S. median age was 38.4 years old in 2019, the median age for Appalachia was 41.3 years old (3). In 2015–2019, 80.7% of the Appalachian population were White, 8.4% were not covered by health insurance, 15.2% of all persons lived in poverty, and 12.8% of the population aged 25 years old and older had less than a high school diploma (3). Only 12% of the population in rural Appalachia identifies as a member of racial or ethnic minority groups, making it less diverse compared to the rest of rural America (25%) (3). Rural Appalachia has reported lower levels of education, employment, and income along with higher levels of poverty and disability than rural areas in the rest of the U.S. (3). Further, rural Appalachian residents experience greater health disparities and also have more limited access to healthcare, mental health services, and social services (4).

Substance use disorders (SUDs)—and opioid use disorder in particular—have become increasingly prevalent and detrimental over the past decade, including in Appalachia. According to the 2020 National Survey on Drug Use and Health, an estimated 40.3 million people ages 12 and older fit the definition for a SUD, with 18.4 million having an illicit drug use disorder and 6.5 million having both an alcohol use disorder and an illicit drug use disorder (5). At the start of 2022, the Centers for Disease Control and Prevention (CDC) released the 2021 provisional drug overdose death counts (5). The reported number of deaths due to drug overdose during the 12-month period ending in June 2021 for the whole country was 98,022 (6). This is over a 30% increase since the 12-month period ending in June 2019 and has worsened in part due to the COVID-19 pandemic (6). The percent change in reported drug overdose deaths in the 12-month period from June 2020 to June 2021 represented an increase of 18.2% (6).

The consequences of SUD can be significant not only for the person experiencing the disease, but also for their families and communities. Research has shown that the rise in drug overdose deaths and drug-related hospitalizations is positively associated with the rise in foster care cases, even after accounting for county-specific socioeconomic and demographic characteristics (7, 8). This association can be observed across the nation. From 2016 to 2017, 39 states experienced an increase in the rate of foster care caseloads, and all five states with the highest opioid overdose rates also experienced these foster care increases (9). West Virginia, a state within Appalachia, specifically had both the highest rate of opioid overdose deaths and the highest foster care rate at 17.8 per 1,000 children in 2017 (9). Further, Appalachia in general has experienced markedly elevated rates of both overdose deaths and foster care entries since 2012 (7, 10).

If a child must be removed from his or her home, social workers will first see if any biological family members could provide a suitable home for the child. Known as kinship care, this approach is intended to lessen the amount of disruption for the child, keep familial ties in place, and allow the biological parents to know their child is with someone they know and trust (11, 12). In 2018, there was an estimated 2.7 million children in the U.S. living in a kinship care situation, and the average age of the kin caregiver was 59 years old (13). Grandparents often serve in the role of kin caregiver (14).

Higher rates of grandparents as the primary caregivers have been observed in Appalachia compared to other parts of the nation (15). This is paralleled by studies indicating that Appalachian states have much higher opioid prescribing rates, high rates of illicit drug use and prescription drug misuse, and higher rates of overdose deaths (10, 16, 17). Young adults between the ages of 25 and 44, key parenting years, are a primary population experiencing SUD, with 53% of opioid overdose deaths in the U.S. in 2020 likewise occurring in this age group (18). In addition, data shows that the most common reasons for kinship care situations include substance use and incarceration, with up to 19% of relative placements due to a mother's substance use, 15% due to a father's incarceration, and 7% due to a mother's incarceration (13). Thus, the rate of grandparents as caregivers could be directly impacted directly by rates of parental substance use and overdose by parents (19). Given evidence suggesting rates of both grandparents as primary caregivers and overdose mortality are elevated in Appalachia, the association between these outcomes could be even stronger in this region compared to others; however, this relationship is understudied.

Multiple factors associated with grandparents serving as primary caregivers of children could contribute to challenges for both grandparents and the children in their care (15). Grandparents caring for grandchildren report more limitations of daily activities, increased depression, lower levels of marital satisfaction, and poorer health (20). In addition, research suggests kinship caregivers in general tend to be older and unemployed (21). Kinship caregivers were found to be four times more likely to have dropped out of high school when compared to non-kinship caregivers and three times more likely to make an annual household income of <$20,000 (21). Approximately 20% of children being raised by their parents were living in poverty in 2012, compared to 25% of children raised by their grandparents (22). Further, teenagers who live with kinship caregivers had almost seven times the risk of pregnancy and two times the risk of substance use than those in foster care (21).

The purpose of this study was to assess the association of drug overdose mortality, as a proxy for SUD, with grandparents serving as primary caregivers of children, and focusing on comparing Appalachian and non-Appalachian regions. We hypothesized that counties with higher overdose mortality rates would have higher rates of grandparents serving as primary caregivers. Compared to non-Appalachian counties, we hypothesized that the rate of grandparents serving as caregivers would be higher and that the relationship between grandparents serving as caregivers and drug overdose mortality would be stronger in Appalachian counties. The findings could inform policies and programs to support grandparents serving as caregivers of children and mitigate the potential impacts of parental SUD on children in kinship care.

## Methods

### Study sample and design

National in scope, this study used a cross-sectional design to identify associations between SUD and grandparents serving as primary caregivers of children. Data encompassed all U.S. counties and identified differences between Appalachian and non-Appalachian counties in relationships between SUD and grandparents as caregivers. Multiple sources of county-level data were combined to create a national-level dataset. Sources included the United States Department of Agriculture (USDA), ARC, U.S. Census (including American Community Survey or ACS), National Center for Health Statistics (NCHS), and County Health Rankings (23–28).

### Data sources and measures

Primary variables of interest included the percent of grandparents as caregivers (dependent) and overdose mortality rates (independent). County-level data on the percent of grandparents serving as primary caregivers of children under 18 years were from 2015 to 2019 ACS 5-year estimates, a demographics survey program implemented annually across all 50 states by the U.S. Census Bureau (28). The NCHS drug poisoning mortality dataset includes an estimated range of drug poisoning deaths per 100,000 at the county-level (26). Estimates are based on data from the National Vital Statistics System multiple cause-of-death mortality files. This dataset was selected to calculate overdose mortality rates as it was the most complete dataset available with most recent available data from 2015. Other available sources had many missing or unreliable data for counties. As the NCHS drug poisoning dataset is an estimated range rather than a singular number, the midpoint of each range was chosen as the overdose mortality rate for each county. Given the interest in comparing between Appalachia and non-Appalachia, classification of counties as Appalachian vs. non-Appalachian was based on guidelines from ARC (24). For purposes of this study, the term counties is used to refer to both counties and independent cities.

Multiple sociodemographic and geographic variables were included as covariates, including median age of the population, percent of the population that is uninsured, percent of the population that is non-Hispanic white, teen birth rate, percent of high school dropouts, and rurality. The U.S. Census provides accurate economic, demographic, and population data on a county-level. Population data from 2018, including the median age of the population and percent of the population that is uninsured, were pulled from this source (25, 29). Data on high school dropout percentages were pulled from 2015 to 2019 ACS 5-year estimates based on population and currently enrolled estimates of individuals aged 15 to 17 years, while data on the percent of the population that is non-Hispanic white were pulled from 2018 County Health Rankings (27, 28). The teen birth rate was pulled from 2018 NCHS data (30). Lastly, the USDA Economic Research Service has established Rural-Urban Continuum Codes (RUCC) for counties using whole numbers 1 through 9 based on population size of metropolitan area (metropolitan counties) or degree of urbanization and adjacency to metro area (nonmetropolitan counties) (23). These RUCC codes were used to classify counties as rural (RUCC 4-9) or non-rural (1-3) for purposes of this study (23). Data ranged from 2015-2019 except of RUCC, which is the most recently available, to explore trends before the COVID-19 pandemic.

### Statistical analysis

All analyses for this study were conducted using SAS 9.4 for Windows. Descriptive statistics such as mean [SD], frequencies, and percentages were reported for all variables of interest from the combined dataset. Key characteristics of counties were examined by Appalachian status *via* Chi-squared tests (rural vs. non-rural) or two-sample *t*-tests (e.g., high school dropout rate, teen birthrate), as appropriate. The three primary hypotheses examined associations between grandparents as caregivers, overdose mortality, and Appalachian vs. non-Appalachian status. Hypothesis 1: counties with higher rates of overdose mortality will have higher rates of grandparents serving as primary caregivers of children under the age of 18. Hypothesis 2: the rate of grandparents serving as primary caregivers of children under the age of 18 will be higher in Appalachian counties than in non-Appalachian counties. Hypothesis 3: the relationship between grandparents serving as primary caregivers of children under the age of 18 and overdose mortality will be stronger in Appalachian counties than in non-Appalachian counties. Hypotheses 1 and 2 were examined individually using bivariate linear regression models with grandparents as caregivers as the dependent variable and overdose mortality (Model 1) or Appalachian status (Model 2) as the independent variable in each model. For Hypothesis 3, a multivariable linear regression model was used that included main effects for both overdose mortality and Appalachian status as well as the interaction between them to see if the relationship between overdose mortality and grandparents as caregivers (Model 3) differed by Appalachian status. Finally, a full multivariable model with all key characteristics as well as the interaction between overdose mortality and Appalachian status was examined in a backwards stepwise fashion to include only significant factors remaining in the final model to interpret these relationships while adjusting for important sociodemographic factors (Model 4).

## Results

### County characteristics

Table 1 presents the sociodemographic characteristics of the 3,142 counties (and independent cities) included in this study. A total of 431 counties (13.7%) were classified as Appalachian, and over 62% were designated rural based on RUCC designation (1). There were no significant differences between Appalachian and non-Appalachian counties in the proportion of rural counties (64 vs. 63%; *p* = 0.7) or mean[SD] high school drop-out percent (5%[4%] vs. 5%[4%]; *p* = 0.5). Compared to non-Appalachian counties, Appalachian counties had a higher mean[SD] teen birth rate per 1000 (27.57[10.44] vs. 23.11[12.07]; *p* < 0.01), median age (42.76[4.20] vs. 41.22[5.56]; *p* < 0.01), and percent of the population that is non-Hispanic white (87.61[12.56] vs. 74.83[20.50]; *p* < 0.01). Non-Appalachian counties had a higher percent of uninsured residents compared to Appalachian counties (11.61[5.17] vs. 10.79[4.06]; *p* < 0.01). Further, Appalachian counties, relative to non-Appalachian counties, had a higher overdose mortality rate per 100,000 (20.41[6.84] vs. 14.65[6.38]; *p* < 0.01) and a higher percent of grandparents serving as primary caregivers of children (51.27[14.31] vs. 45.80[18.06]; *p* < 0.01).

**Table 1:** Sociodemographic characteristics of U.S. counties, overall and by Appalachian status.

	**All (*****N*** = **3,142)**		**Appalachian (*****N*** = **431)**		**Non-Appalachian (*****N*** = **2,711)**		
	***N***	**%**	***N***	**%**	***N***	**%**	***p*** **-value**
Non-rural^*^	1,166	37.11	156	36.19	1,010	37.26	0.7
Rural^*^	1,976	62.89	275	63.81	1,701	62.74	
	**Mean**	**SD**	**Mean**	**SD**	**Mean**	**SD**	***p*** **-value**
Teen birth rate per 1,000	23.72	11.95	27.57	10.44	23.11	12.07	< 0.01
Percent high school dropout	4.05	4.76	4.21	4.59	4.03	4.79	0.5
Percent non-Hispanic white	76.58	20.09	87.61	12.56	74.83	20.50	< 0.01
Percent uninsured	11.50	5.04	10.79	4.06	11.61	5.17	< 0.01
Median age (in years)	41.43	5.42	42.76	4.20	41.22	5.56	< 0.01
Overdose mortality rate per 100,000	15.44	6.74	20.41	6.84	14.65	6.38	< 0.01
Percent of grandparents as caregivers	46.56	17.69	51.27	14.31	45.80	18.06	< 0.01

* RUCC code 1–3 = non-rural; 4–9 = rural.

### Association between grandparents as caregivers and overdose mortality

Table 2 presents the results of the bivariate linear regression model (Model 1) examining the relationship between grandparents as primary caregivers of children with overdose mortality. The percent of grandparents as caregivers increased as the overdose mortality rate increased (*p* < 0.01). For every 1% increase in the overdose mortality rate, the percent of grandparents as caregivers increased by 35%. Similarly, Table 2 presents the results of the other regression models (Models 2–4). Model 3 examines the relationship of overdose mortality, Appalachian status, and the interaction between these variables on grandparents as caregivers. There was a statistically significant interaction between overdose mortality rate and Appalachian vs. non-Appalachian counties (*p* = 0.01), indicating that the relationship between overdose mortality rate and the rate of grandparents as caregivers is different for Appalachian counties and non-Appalachian counties. In Appalachian counties, for every 1% increase in the overdose mortality rate, the percent of grandparents as caregivers increased by 56% compared to non-Appalachian counties, where every 1% increase in the overdose mortality rate, the percent of grandparents as caregivers increased only by 24%. Model 4 presents a fuller model that adjusts for county-level sociodemographic characteristics including percent of population uninsured, percent of non-Hispanic white population, median age, rural vs. non-rural, percentage of high school dropout, and teen birth rate. After adjusting for those characteristics, the interaction between overdose mortality and Appalachian vs. non-Appalachian counties was no longer significant (*p* = 0.4). Characteristics more strongly associated with the rate of grandparents as caregivers included the percent of non-Hispanic white (*p* < 0.01), rurality (*p* < 0.01), percent uninsured (*p* = 0.02), and teen birth rate (*p* < 0.01).

**Table 2:** Results of bivariate and multivariable linear regression models examining county-level associations with grandparents as primary caregivers of children.

	**B**	**SE**	***p*-value**	**Model**
Overdose mortality rate per 100,000	0.35	0.05	<0.01	1
Appalachian vs. non-Appalachian	5.47	0.91	<0.01	2
Overdose mortality rate per 100,000 (A)	0.24	0.05	<0.01	3
Appalachian vs. non-Appalachian (B)	−2.57	2.79	0.4	
A^*^B interaction	0.33	0.13	0.01	
Overdose mortality rate per 100,000 (A)	0.09	0.05	0.1	4
Appalachian vs. non-Appalachian (B)	−0.89	2.62	0.7	
A^*^B interaction	0.11	0.13	0.4	
Percent uninsured	0.16	0.07	0.02	
Percent non-Hispanic white	0.13	0.02	<0.01	
Median age (in years)	0.08	0.06	0.2	
Rural vs. non-rural^*^	5.47	0.69	<0.01	
Percent high school dropout	−0.06	0.06	0.3	
Teen birth rate per 1,000	0.42	0.03	<0.01	

* RUCC code 1–3 = non-rural; 4–9 = rural.

B, Beta estimate from linear regression model.

SE, Standard error from linear regression model.

Dependent outcome for all models = Percent of grandparents as caregivers.

## Discussion

This study explored the association between drug overdose mortality, as a proxy for SUD, and grandparents serving as primary caregivers of children in Appalachian and non-Appalachian regions. It adds to the existing body of literature documenting the multi-generational impacts associated with SUD, particularly in Appalachia. Overall, counties with higher overdose mortality rates had greater rates of grandparents as primary caregivers. This association parallels trends that have been observed nationally in relation to overdose hospitalizations and mortality as well as increases in foster care placements (7). Although some children are cared for by their grandparents through the formal foster care system, the majority of grandparents are serving as caregivers outside the system (31). Those that are functioning outside of the system may have less access to support services such as caregiver subsidies, parent training, peer support, and respite care (21).

Consistent with our hypothesis, rates of grandparents as primary caregivers were greater in Appalachian compared to non-Appalachian counties. In a multivariable model including the interaction between overdose morality and Appalachian status, we found that the rates of grandparents as caregivers were greater in Appalachian counties than in non-Appalachian counties. This is consistent with a previous study looking at the intersection of poverty, geography and grandparents as caregivers (15). Grandparents residing in Appalachian counties more negatively impacted by overdose mortality are more likely to be primary caregivers than grandparents residing in non-Appalachian counties with high overdose mortality. This discrepancy could speak to a few different important factors. There might be a lack of other family members to support children, including spouses, leading to grandparents formally or informally filling this role. For those acting as caregivers through the formal foster care systems, kinship fostering is preferred to non-kinship. Though this provides less disruption to the child, data show that family members often have less resources than non-kin (21). There also could be cultural differences, where strong familial ties that commonly characterize Appalachia and rural areas (32) have an impact on grandparents, leading more to answer the call to support their grandchildren in times of need.

When including multiple variables that are associated with greater overdose mortality and grandparents as caregivers, Appalachian county was no longer a significant predictor of grandparents as caregivers (7, 21, 22). This change is not that surprising considering the factors of uninsured, non-Hispanic white population, rurality, higher percentage of high school dropout, and higher teen birth rate are all also strongly associated with Appalachia (3). In other words, these factors are often more prevalent in many communities in Appalachia and are likely driving the differences observed between Appalachian and non-Appalachian counties.

The COVID-19 pandemic has been attributed to higher rates of overdose deaths, with the highest rates recorded nationally in 2021. This increase can be expected to continue to strain the formal and informal foster care system. Based on our study, there will likely be even greater numbers of grandparents caring for grandchildren in the coming years as a consequence of overdose deaths in individuals during the COVID-19 pandemic (33). Data demonstrate the importance of familial caregiving, but policies and programs will be needed to better support families, grandparents in particular, to best care for children impacted by SUD. The need for such supports could be particularly acute among grandparents providing care outside of the foster care system through education and training, support groups, case management, and financial and transportation support (32). Generations United, whose mission is to improve the lives of children, youth, and older people through intergenerational collaboration, public policies, and programs for the enduring benefit of all (34), has suggested a reform to federal child welfare financing to support children, parents, and grandparent caregivers such as kinship navigation, substance use treatment and prevention services, mental health services and in-home supports (35). Kinship navigation programs provide opportunities for grandparents to access information about the services that are available for them, and these types of programs could be especially beneficial for rural and Appalachian communities where resources and services may be limited (21, 31). Parenting interventions targeting grandparents, both in the system and outside of it, can improve caregivers' parenting competency, reduce parental stress, and advance child wellbeing (36).

### Limitations

This study has several limitations that should be considered. The cross-sectional design limits the ability to draw causal inferences about the association between grandparents serving as caregivers of children and drug overdose mortality, including the direction of the association. This study used drug overdose mortality as a proxy for SUD given challenges with accessing reliable, publicly available data on substance use at the county-level. Similarly, this study relied on self-reported data from ACS on grandparents serving as caregivers, whether within or outside of the formal foster care system. Future research should integrate data from the foster care system to better understand the system-specific impacts of SUD. In addition, multiple publicly available data sources were combined for this study. The time points across sources do not always align; however, when available, 5-year estimates were used to be more reflective of trends during the overall time frame of the study.

### Public health implications

This study found that counties with higher rates of overdose mortality had greater numbers of grandparents serving as primary caregivers of children and that there were higher rates of grandparents as caregivers in Appalachian counties compared to non-Appalachian. Programs and policies are needed to support grandparents as they provide crucial caregiving for children impacted by parental SUD.

## Data availability statement

Publicly available datasets were analyzed in this study. This data can be found here: https://www.census.gov/programs-surveys/acs; https://www.countyhealthrankings.org/explore-health-rankings/rankings-data-documentation; https://data.cdc.gov/NCHS/NCHS-Drug-Poisoning-Mortality-by-County-United-Sta/pbkm-d27e; https://www.census.gov/data.html.

## Author contributions

KB lead author, developed the concept, and contributed to the writing and editing. SM lead the writing of the manuscript and supported the analysis. AM wrote the introduction and edited the draft. MF performed analysis, wrote methods and results sections, and reviewed the manuscript. AW lead the analysis, wrote methods and results sections, and edited the draft. MM senior researcher, reviewed the manuscript, and edited. All authors contributed to the article and approved the submitted version.
